# A Capillary-Evaporation Micropump for Real-Time Sweat Rate Monitoring with an Electrochemical Sensor

**DOI:** 10.3390/mi10070457

**Published:** 2019-07-07

**Authors:** Xiao-Ming Chen, Yong-Jiang Li, Dan Han, Hui-Chao Zhu, Chun-Dong Xue, Hsiang-Chen Chui, Tun Cao, Kai-Rong Qin

**Affiliations:** 1 School of Optoelectronic Engineering and Instrumentation Science, Dalian University of Technology, Dalian 116024, China; 2 School of Biomedical Engineering, Dalian University of Technology, Dalian 116024, China

**Keywords:** capillary–evaporation effect, electrochemical principle, micropump, sweat collection, sweat rate

## Abstract

Sweat collection and real time monitoring of sweat rate play essential roles in physiology monitoring and assessment of an athlete’s performance during exercise. In this paper, we report a micropump for sweat simulant collection based on the capillary–evaporation effect. An electrochemical sensor is integrated into the micropump, which monitors the flow rate in real-time by detecting the current using three electrodes. The evaporation rate from micropore array, equivalent to the sweat rate, was theoretically and numerically investigated. The designed micropump yields the maximum collection rate as high as 0.235 μL/min. In addition, the collection capability of the micropump was validated experimentally; the flow rate through the microchannel was further detected in real-time with the electrochemical sensor. The experimental maximum collection rate showed good consistency with the theoretical data. Our proposed device shows the potential for sweat collection and real-time monitoring of sweat rate, which is a promising candidate for being a wearable platform for real-time physiology and performance monitoring during exercise.

## 1. Introduction

Sweat is an essential physiological biofluid that contains many important biomarkers (e.g., electrolyte, proteins, glucose, and lactate) related to health conditions such as dehydration, physical fatigue, mental stress and disease [1,2,3,4]. For this reason, sweat content analysis has drawn attention to sweat-related physiological monitoring and non-invasive disease diagnosis, providing information about the health and well-being of subjects, especially during sports activities [2,5,6,7,8]. Due to the secretion and reabsorption nature of sweat production within the sweat gland and duct, sweat composition concentrations vary with the changes in sweat rate [9,10,11]. In this respect, continuous collection of sweat and real-time monitoring of sweat rate are necessary prerequisites for sweat analysis, which shows potential in physiological studies aimed at diagnosing disease and optimizing performance.

Since the 1930s, sweat collectors have been developed by numerous collection methods for whole-body and local sweat analysis [9,12]. Whole-body sweat collection method has been confined to the laboratory [9], inappropriate for a portable sweat sensor. In the past decades, microfluidics and MEMS (Micro-Electro-Mechanical Systems) have shown advantages in fluid manipulation, biosensing and integration ability of multiple functional modules, which greatly contributed to the development of local sweat sensing devices. Surface tension and capillary forces were widely applied to drive and collect sweat using gauze pads, highly absorbent pads and well-designed configuration of microchannels [12,13,14]. Microheaters and temperature regulator have also been applied to accelerate the sweat evaporation rate [15,16]. However, these methods introduce complexity into the system and are difficult to realize a long-term continuous flow of sweat. Inspired by the transpiration in plants, several researchers [16,17,18] reported micropumps for passively generating a prolonged and continuous flow for sweat collection by using the combination of capillary and evaporation effects, revealing the advantages of simple operation, good flexibility and low fabrication cost. Nevertheless, these sweat collectors lack the real-time sensing and monitoring abilities for application in sweat sensing devices.

Sweat rate achieved from sweat collectors needs to be quantified for sweat content analysis. Initial studies on sweat rate were performed by determining the change in body mass, which was considered as a gold standard [9]. Recently, numerous sweat rate sensors have been realized based on electrical [10,19,20], electrochemical [1,2] and optical principles [21]. Lien et al. demonstrated a flow rate sensor based on the change in optical transparency of the optical fiber cantilever induced by flowing fluid. Francis et al. [10] and Yang et al. [20] reported the digital droplet method to characterize the sweat rate with variation in electrical conductivity. While these sensors have high sensitivity, the fabrication and integration procedures are complex. There are other sweat sensing devices using image analysis [22], humidity sensors [23], ventilated capsule technique [24] and calorimetric method [25,26,27]. However, real-time sensing of sweat rate still faces severe challenges, including complicated peripheral detection system, the capacity of real-time monitoring and the integration with active sweat collectors. Therefore, a microfluidic device capable of collecting long-term continuous sweat flow and integrating an on-chip sensor for real-time sweat rate monitoring is highly required.

Towards this goal, we present a novel micropump with an electrochemical sensor capable of actively collecting sweat and real-time monitoring of sweat rate. The device can drive and collect sweat using the combination of capillary and evaporation effects. In addition, we demonstrated an electrochemical sensor integrated into the designed micropump used for monitoring the sweat/evaporation rate in real-time. Using a theoretical evaporation model and numerical simulation, the maximum evaporation rate was studied. The effectiveness of the device, the capacity of the sweat collection, and real-time flow rate monitoring were experimentally validated.

## 2. Materials and Methods

### 2.1. Microfluidic Device Fabrication

**Figure 1:**
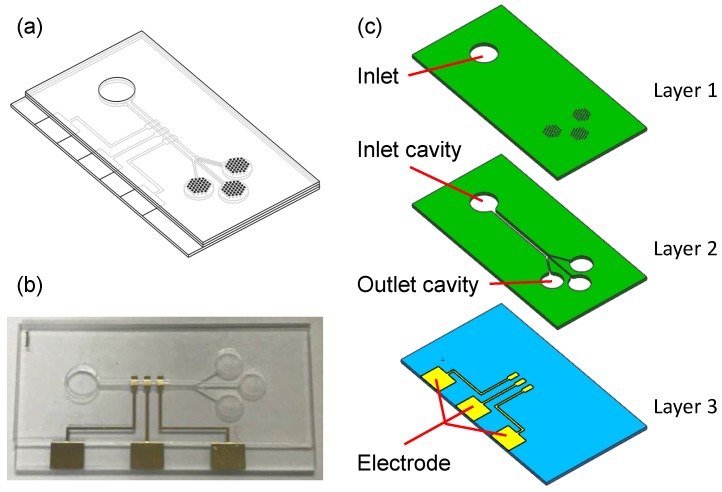
(**a**) Schematic of the proposed microfluidic device; (**b**) an image of the real device; and (**c**) structural diagram of the device consisting of three layers.

Figure 1 shows the schematic of the proposed microfluidic device, which is made of a three-layer structure. The top layer (40 mm × 20 mm × 0.25 mm) of polymethyl methacrylate (PMMA) contains the inlet (5 mm in diameter) and three micropore arrays (250 μm in diameter and 500 μm in the interval) correspondingly connecting to the inlet cavity (5 mm in diameter) and outlet cavity (4.1 mm in diameter) of the middle layer (40 mm × 20 mm × 0.25 mm). A microchannel in the middle layer connects the inlet cavity and outlet cavity. The upper two layers are fixed onto a glass layer (40 mm × 22.5 mm × 1.5 mm), on which an electrochemical sensor is patterned beneath the microchannel. The three layers are stuck to each other with the double-sided adhesive film (SG-D02, Wenhao Co. Ltd., Suzhou, China). The electrochemical sensor, proposed for quantitatively measuring the evaporation rate equivalent to the sweat rate, consists of a triple-electrode (2 mm × 1 mm) system, being separated by identical gaps of 1 mm. Figure 1c shows the schematic of the printed electrodes used for electrochemical sensing: working electrode (WE), reference electrode (RE), and counter electrode (CE). The three electrodes are linked with three big exposed ones (5 mm × 4 mm) for connecting to peripheral measurement equipment. The electrochemical sensor is fabricated using a high vacuum magnetron sputtering coating system (JP-200, Technol Science Co. Ltd., Beijing, China). All the electrodes are made of inert gold to improve the sensitivity and to reduce the electrode drift.

For an applied wearable sweat collector, the inlet will be attached to the skin surface through an absorbent film. Hence, the sweat can be absorbed by the film and transported into the inlet cavity. Due to the capillary effect of the microchannel and the evaporation effect of micropores, the sweat will be driven into the microchannel and transmitted through the sensing area towards the outlet cavity. The continuous flow of sweat yields the variation in current detected by the electrochemical sensor, which can be applied to characterize the flow rate. In this study, we focus on the microfluidic aspect of sweat collection and the electrochemical sensor for monitoring flow/evaporation rate. A wearable and flexible sweat sensing device will be concerned in future studies.

### 2.2. Theoretical and Numerical Analysis of Evaporation Rate

#### 2.2.1. Evaporation Theory

**Figure 2:**
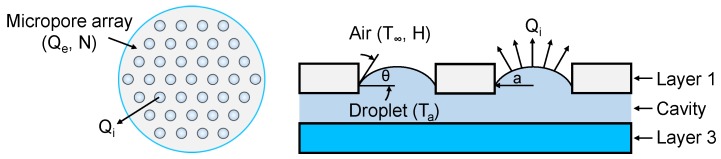
Schematic of sessile droplets evaporated from the arrayed micropores.

In the device, the capillary–evaporation effect of micropores at the outlet drives a continuous flow through the microchannel (see Figure 1), which leads to a volumetric evaporation rate $Q_{e}$. To obtain the total evaporation rate $Q_{e}$, we first consider the evaporation rate from a single micropore $Q_{single}$. Based on the evaporation theory [16], the evaporation rate of a sessile droplet as shown in Figure 2 can be expressed as [28]

(1) $$Q_{single}=\frac{dV}{dt}=-2\pi \frac{DM}{\rho }\left[c\left(T_{a}\right)-Hc\left(T_{\infty }\right)\right]F\left(\theta \right)a,$$

where *V* is the droplet volume, *t* is the time, *D* is the diffusion coefficient in air at ambient temperature, *H* is the relative humidity of the ambient air, *M* and ρ are the molar mass and the density of the vapor–liquid, and *a* is the radius of the bounding circle (Figure 2). $T_{a}$ and $T_{\infty }$ are the temperature of the droplet and the ambient air, respectively. Accordingly, $c(T_{a})$ and $c(T_{\infty })$ the molar concentrations of saturated vapor at the corresponding temperature. F(θ) in Equation (1) is a function of the contact angle θ (Figure 2), which is given by [29]

(2) $$F\left(\theta \right)=\left\{\begin{array}{cc} 2/\pi , & \;\theta =0\\ \left(0.6366\theta +0.0959\theta^{2}-0.0614\theta^{3}\right)/\sin\theta , & \;0<\theta \leq 0.175\\ \left(0.00008957+0.6333\theta +0.166\theta^{2}-0.08878\theta^{3}+0.01033\theta^{4}\right)/\sin\theta , & \;0.175<\theta \leq \pi \end{array}\right.$$

Note that Equations (1) and (2) denote the evaporation rate of a sessile droplet from a single micropore. In the case of micropore array in our device (Figure 1), the interdroplet interaction from neighboring micropores reduces the evaporation rate from a local micropore. To recognize this concern, the evaporation rate of the *i*th pore in an array of *N* pores can be corrected by introducing the evaporation correction factor $\eta_{i}$, which is expressed as

(3) $$Q_{i}=\eta_{i}Q_{single}.$$

Herein, the evaporation correction factors $\eta_{i}$ of the droplet array are derived based on the approximate point source method, which satisfies a set of *N* linear equation

(4) $$\eta_{i}+\sum_{j=1,j\neq i}^{N}\left(\eta_{j}\frac{a_{j}}{\left|r_{i}-r_{j}\right|}\right)=1,\;i=1,2,\cdots ,N,$$

where $a_{j}$ is the radius of *j*th droplet and $\left|r_{i}-r_{j}\right|$ denotes the distance between *i*th and *j*th droplet. In the case of a uniform micropore array [30], the average evaporation correction factor $\bar{\eta }$ is calculated as

(5) $$\bar{\eta }=\frac{1}{N}\sum_{i=1}^{N}\eta_{i}.$$

The total evaporation rate $Q_{e}$ is thus given by

(6) $$Q_{e}=N\bar{\eta }Q_{single}.$$

With Equations (1), (5) and (6), the analytical evaporation rate can be calculated, which was compared with both experimental and simulation results.

#### 2.2.2. ANSYS Numerical Simulation

The maximum evaporation rate was characterized quantitatively using the engineering simulation software ANSYS. The 3-D simulation was conducted with the same dimensions of the microfluidic chip (see Figure 1). The fluid field of the microchannel was extracted as the simulation domain, which was divided into 446,349 elements and 407,375 nodes. The meshing result was imported into ANSYS Fluent 18.0. The modules including Viscous–Laminar model, Evaporation–Condensation model and Schiller–Naumann model were adopted to obtain a steady solution of flow rate. In the Evaporation–Condensation model, evaporation effect of micropores drives the fluid flow to the outlet cavity until saturated vapor pressure is reached, corresponding to the steady status. In this case, the evaporation rate was equivalent to the maximum flow rate for sweat collection. The maximum flow rate was obtained at a temperature of 20 ${}^{\circ }$C. The fluid used in the simulation was water with the density of 1000 kg/m${}^{3}$.

### 2.3. Characterization of Electrochemical Sensor

#### 2.3.1. Electrochemical Principle for Flow Rate Monitoring

The current response of an electrochemical detector is governed by the convection and diffusion towards the electrode surface and ruled by the hydrodynamic condition of the flow. A general current response for mass-transport-controlled reactions [31] is

(7) $$i_{l}=nFADC/\delta ,$$

where *n* is the number of electrons transferred per molecule, *F* is the Faraday constant, *A* is the electrode area, *D* is the diffusion coefficient, and *C* is the bulk concentration of the electroactive species. δ denotes the thickness of the diffusion layer. According to the Nernst approximate approach, δ is empirically related to the average flow rate *Q*, which is expressed by

(8) $$\delta =B/Q^{\alpha },$$

where *B* and α constants depend on the specific characteristics of the hydrodynamic conditions. By taking into account the hydrodynamic properties of the flowing solution, the limiting current of electrodes under steady-state condition is derived by solving the three-dimensional convective diffusion equation. For the proposed thin-layer electrodes in this study, the limiting current in Equation (7) can be expressed as

(9) $$i_{l}=1.47nFC{\left(DA/b\right)}^{2/3}Q^{1/3},$$

where *A* is the electrode area and *b* is the microchannel height. It can be seen from Equation (9) that the limiting current $i_{l}$ is dependent on the volume flow rate *Q*, which is proportional to the one third power of *Q*. Hence, the variation in *Q* results in the variation in $i_{l}$, which can be measured with an electrochemical workstation for flow rate detection.

#### 2.3.2. Experimental Validation

**Figure 3:**
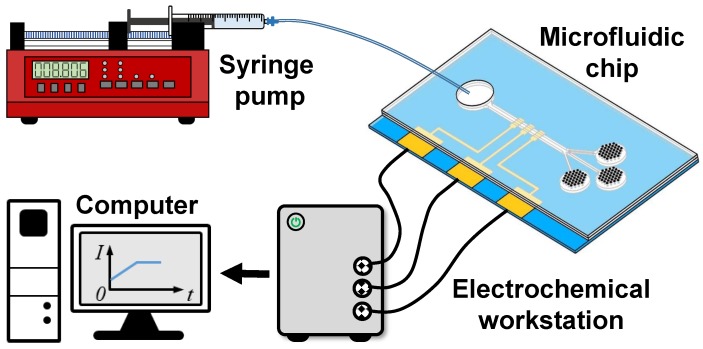
Schematic diagram of the experimental system.

An experimental system (Figure 3) was constructed to validate the effectiveness of the proposed micropump for sweat collection and real-time sweat rate monitoring. The system consisted of a syringe pump, the microfluidic chip, an electrochemical workstation (CHI760E, CH, Shanghai, China) and a computer. As shown in Figure 3, a syringe fixed to a syringe pump (NE-1000, New Era, NY, USA) was connected to the inlet of the microfluidic chip. The syringe was filled with sodium chloride solution (NaCl 0.9% *w*/*v*) to simulate sweat. The three large electrodes of sensor integrated on the chip were connected to the electrochemical workstation. The detected current signal of the electrochemical workstation was transmitted to the computer for further analysis.

Before validating the designed device, we first calibrated the electrochemical sensor using the designed experimental system (Figure 3). NaCl solution was injected via a syringe pump at a predefined flow rate of 0.2 μL/min, 0.4 μL/min, 0.8 μL/min and 1.2 μL/min, which can simulate perspiration of human skin at different intensities of physical activity. In each case, the current corresponding to a steady flow rate was recorded and used to obtain the calibration curve of current as a function of flow rate.

After calibration, the capacity of sweat collection and real-time sweat rate monitoring was validated. A drop of NaCl solution was introduced into the inlet cavity, which was then covered with a PDMS film to avoid evaporation. The NaCl solution was thus driven by spontaneous evaporation of micropore arrays. The time evolution of the current was detected to characterize the flow rate in real time. In all cases, at least three trials were performed. All experiments were conducted at room temperature (20 ${}^{\circ }$C).

## 3. Results

### 3.1. Maximum Evaporation Rate

To characterize the capacity of the designed micropump for sweat collection, we calculated the maximum evaporation rate based on the theoretical model in Section 2.2.1. With the dimensions of the microchannel (see Section 2.1), the evaporation rate $Q_{e}$ in Equation (6) was calculated. The three micropore arrays had a total evaporation rate of 0.241 μL/min at the temperature of 20 ${}^{\circ }$C.

**Figure 4:**
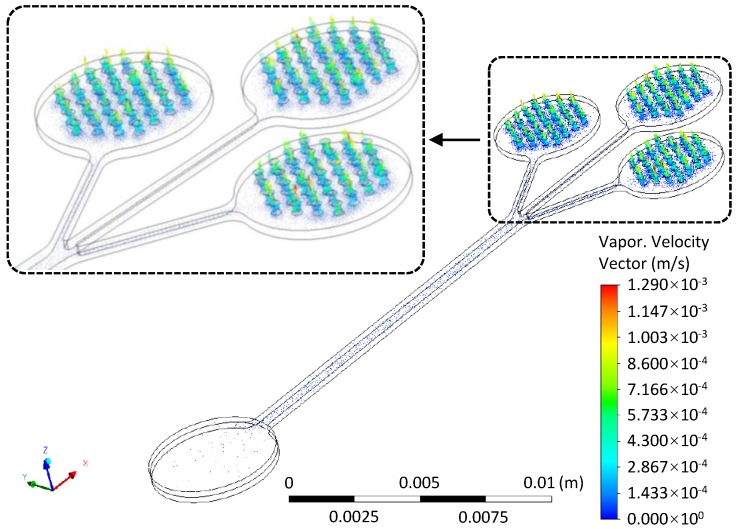
Vector of evaporation velocity at the whole fluid domain and at the outlet cavities (upper left).

Numerically, the evaporation rate under the steady condition was investigated. Figure 4 shows the vector of evaporation velocity within the microchannel and the outlet cavities. The maximum evaporation velocity appeared at the micropore arrays. The average mass rates of the micropore arrays were: −1.324$\times 10^{-9}$ kg/s, −1.332$\times 10^{-9}$ kg/s and −1.328$\times 10^{-9}$ kg/s. Herein, negative sign denotes the direction of evaporation. Thus, the total evaporation rate was 0.239 μL/min, which is consistent with the theoretical result.

### 3.2. The Capacity of Sweat Collection

**Figure 5:**
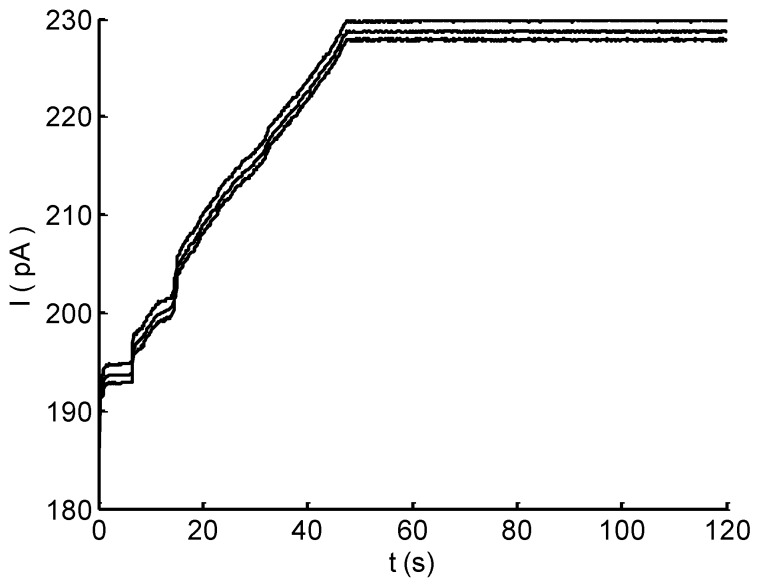
Time evolution of electrode current after introducing a drop of NaCl solution into the inlet cavity.

We validated the collection capacity of micropump by introducing a drop of NaCl solution into the inlet cavity. The solution can immediately fill the microchannel due to its capillary effect. From the evolution of electrode current in Figure 5, the sharp increase current, in the beginning, corresponded to the quick filling. Afterwards, the approximate linear increase in current demonstrated the increase in the evaporation effect of micropores. The current leveled off after ∼50 s and remained at a constant level in the recording 120 s. The result reveals that the designed micropump could drive and transport sweat simulant using the capillary effect of microchannel followed by the evaporation effect of micropores, resulting in a continuous fluid through the sensing area for real-time flow rate monitoring.

### 3.3. Calibration of the Flow Rate Sensor

**Figure 6:**
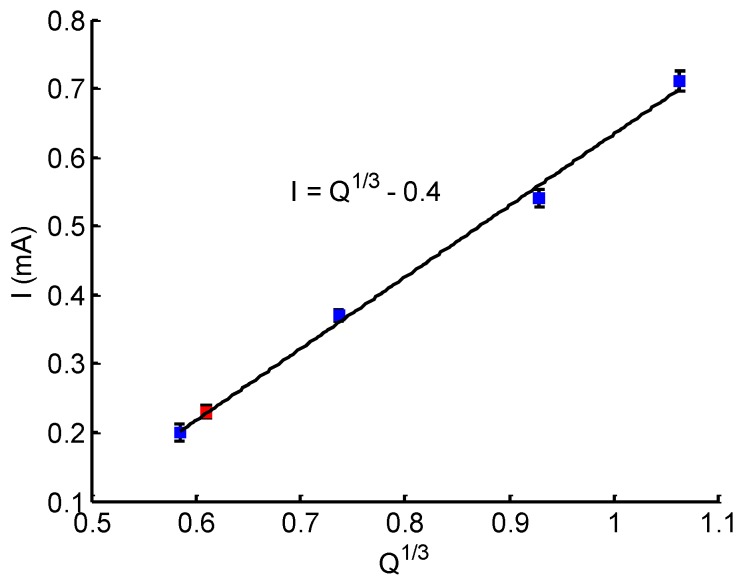
Calibration curve of the electrode current *I* as a function of the flow rate.

Before using the sensor for measurement, it was calibrated. As indicated in Figure 5, a steady evaporation rate resulted in a constant current. Therefore, the maximum current corresponding to a flow rate was applied for calibration. Figure 6 shows the calibration curve. The flow rate of the syringe pump ranged from 0.2 μL/min to 1.2 μL/min, and the corresponding maximum current in each case was detected. The calibration data confirm that the electrochemical sensor was working as expected; the current *I* was linearly related to $Q^{1/3}$, as indicated in Equation (9).

The experimental data were fitted with a formula similar to Equation (9), which is given as

(10) $$I=KQ^{1/3}+i_{0},$$

where *I* is the detected current, $i_{0}$ denotes a baseline offset and *K* is the slope coefficient. With the detected current at the different flow rate, we obtained a calibration curve with K≃1 and $i_{0}≃0.4$. Based on Equation (10), the evolution of flow can be monitored in real time by detecting the electrode current.

### 3.4. Real-Time Monitoring of Flow Rate

**Figure 7:**
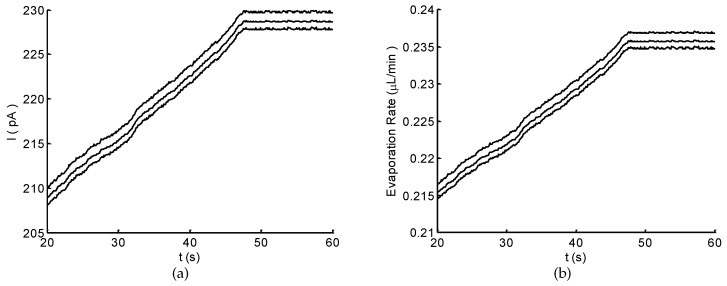
Measurement results of the detected current (**a**); and the corresponding evaporation rate (**b**).

Figure 7a shows an example of the current detected using the electrochemical sensor. The NaCl solution in the inlet cavity was driven and transported by the capillary effect of microchannel and spontaneous evaporation of micropores. As shown in Figure 7a, the current linearly increased until it reached a steady state. Using the calibration curve (Figure 6 and Equation (10)), the corresponding evaporation rates were obtained (Figure 7b), showing a similar tendency as the current. The evaporation effect gradually enhanced until it reached the maximum. This result demonstrates that, under the spontaneous evaporation, a duration of ∼50 s was needed for achieving the maximum evaporation rate. Similar but not identical responses were observed for different trials. For the three trials, the average of maximum evaporation rate was 0.235 μL/min, showing a good agreement with those of the theoretical model and the numerical simulation.

## 4. Discussion

Personalized healthcare and physiology monitoring could be achieved through real-time analysis of biochemical markers and biophysical factor in biofluids. Conventionally, blood is used as a gold standard for illness diagnosis and health examination. This approach is an invasive procedure and only provides information at a specific time. Sweat is an alternative that is easily accessible and contains rich useful information about physiological conditions. Therefore, in the past decade, sweat-based sensing devices have been extensively explored with the advancement in microfluidic and wearable technologies [1,4,12]. Nevertheless, there is still an increasing demand for a high-efficiency sweat collector capable of real-time sweat rate monitoring, which is essential for further sweat content analysis. To this end, we have proposed a microfluidic micropump for sweat collection and real-time sweat rate monitoring. The experimental results (see Section 3.4) demonstrate that the micropump can effectively collection sweat at a maximum rate of ∼0.24 μL/min, and that the flow rate can be monitored in real time with the integrated electrochemical sensor (Figure 7b).

The micropump for sweat collection in this study (see Figure 1) was designed using the capillary–evaporation effects inspired by transpiration in plants [16,17,30]. As indicated in the experimental results (Figure 5), the capillary of microchannel can quickly drive a flow that immediately (less than 2 s) fills the microchannel. Then, the following evaporations of micropores increasingly enhance and drive a continuous flow, showing a high driving efficiency for sweat collection. This driving method of the micropump reveals advantages over those based on capillary and evaporation effects [18,32]. Firstly, the driving forces are high. In addition to the capillary, the evaporation of micropores can be two orders higher than that induced from a macroscale surface [17,33]. Secondly, the evaporation rate equivalent to sweat collection rate is relatively high. The flow rate of evaporation is proportional to the evaporation area. With three micropore arrays (total area of ∼40 mm${}^{2}$), the maximum evaporation rate of ∼0.24 μL/min was achieved. This value is much higher than previously reported micropumps [14,16,18] (flow rate from 0.85 μL/min to 1.32 μL/min for evaporation area from 300 mm${}^{2}$ to 680 mm${}^{2}$) even with electrical fan (3.02 μL/min for 680 mm${}^{2}$) and heater (0.12 μL/min for 25 mm${}^{2}$). Moreover, the micropump is passive and without any external power supply, heater or electric fan. This not only reduces the difficulty in design and fabrication, but also seems promising for a sweat sensing device. It should be noted that the evaporation rate was theoretically and experimentally studied at the temperature of 20 ${}^{\circ }$C. We kept the temperature consistent to mutually validate the results. However, for the ultimate application of the sweat sensing device, body surface temperature can enhance the evaporation rate and further the flow rate [15,16,18]. Moreover, many factors, such as number of pores and pore geometry, can be optimized to improve the collection capacity of the micropump.

Sweat rate is a critical parameter of sweat composition monitoring since analyte concentrations vary with the sweat rate due to the disease, exercise intensity and environmental conditions [11,12]. Herein, we integrated an electrochemical sensor (see Figure 1) into the micropump for monitoring the flow rate in real time. The sensor with three electrode reveals a simple structure and easy fabrication. It can be easily integrated into the micropump for realizing an on-chip flow rate monitoring. Besides, the patterned sensor is flexible which can be applied in a wearable device. The sensor characterizes the flow/sweat rate based on the electrochemical principle, which transduces the flow rate to a current signal (see Section 2.3.1). As such, the sensor can monitor the flow rate in real time by detecting the current evolution (Figure 7b). In addition, the sensitivity and measuring range of the sensor depend on the detection electrochemical workstation, which are limitations for some commercial and academic flow sensors [10,19].

The proposed sweat sensing device has the advantages of high driving efficiency, real-time monitoring, simple implementation, and easy-to-use. However, this study mainly focused on the microfluidic aspects for sweat collection and real-time flow rate monitoring. To be able to apply it as a wearable sweat sensor, at least two points need to be improved. One is the flexibility of fabrication materials. A flexible material, such as the polyethylene terephthalate (PET) [16], should be applied to replace PMMA (see Section 2.1) so that the device can closely attach to the skin for sweat collection. The other point is ambulatory. The proposed sensor is integrated into the micropump while a peripheral workstation is needed for detection. Efficient integration of detection system, wireless and powering components should be improved for ambulatory monitoring [12]. These aspects will be further investigated in the future study, to realize a flexible and wearable sweat sensing device for heath care and the monitoring of athletic performance.

## 5. Conclusions

In this study, we demonstrated a versatile microfluidic platform for sweat collection and real-time sweat rate monitoring. The proposed device can effectively drive and collect sweat simulant to the sensing microchannel based on the capillary–evaporation effect. Simultaneously, the sweat/evaporation rate could be measured in real-time with the three-electrode electrochemical sensor.The capacities of collection and real-time flow rate monitoring were experimentally validated. The maximum evaporation rate of the experimental result is consistent with theoretical and numerical results, revealing the effectiveness of the device. This proposed micropump shows the potential to be further developed into a wearable sweat collector for the real-time monitoring of sweat rate to evaluate health or athletic status.
